# Dietary beet pulp decreases taurine status in dogs fed low protein diet

**DOI:** 10.1186/s40781-016-0112-6

**Published:** 2016-08-02

**Authors:** Kwang Suk Ko, Andrea J. Fascetti

**Affiliations:** 1 Department of Nutritional Science and Food Management, Collage of Science & Industry Convergence, Ewha Womans University, 52 Ewhayeodae-gil, Seodaemun-gu, Seoul, 120-750 Korea; 2 Department of Molecular Biosciences, School of Veterinary Medicine, University of California Davis, One Shield Ave, Davis, CA 95616 USA

**Keywords:** Taurine deficiency, Bile acid excretion, Fiber, Dogs, Dilated cardiomyopathy

## Abstract

**Background:**

It is known that large dogs who are fed lamb and rice diets are at increased risk to develop taurine-deficiency-induced dilated cardiomyopathy. Since dogs obligatorily conjugate bile acids (BA) with taurine, we determined whether rice bran (RB) or other fibers (cellulose; CL, beet pulp; BP) would affect BA excretion and/or the taurine status of dogs.

**Results:**

Eighteen medium/large mixed-breed dogs were given purified diets containing CL, BP, or RB for 12 weeks. Taurine concentrations in plasma and whole blood were significantly decreased at week 12. The BP group, compared to the CL or RB groups, showed significantly lower taurine concentrations in plasma (6.5 ± 0.5 vs 20.4 ± 3.9 and 13.1 ± 2.0 μmol/L, respectively, *P* < 0.01, mean ± SEM) and in whole blood (79 ± 10 vs 143 ± 14 and 127 ± 14 μmol/L, respectively, *P* < 0.01), lower apparent protein digestibility (81.9 ± 0.6 vs 88.8 ± 0.6 and 88.1 ± 1.2 %, respectively, *P* < 0.01), and higher BA excretions (5.6 ± 0.1 vs 3.4 ± 0.5 and 3.4 ± 0.4 μmol/g feces, respectively, *P* < 0.05) at week 12.

**Conclusions:**

These results do not support the hypothesis that RB is likely to be a primary cause of lamb meal and rice diets, increasing the risk of taurine deficiency in large dogs. However these indicate that BP may contribute to a decrease taurine status in dogs by increasing excretion of fecal BA and decreasing protein digestibility, thus decreasing the bioavailability of sulfur amino acids, the precursors of taurine.

## Background

It is known that dogs, under normal dietary conditions, synthesize taurine via the activities of two key enzymes, cysteine dioxygenase and cysteine sulfinic acid decarboxylase [1]. Taurine is synthesized from its precursor cysteine, resulting in sufficient quantities to meet their metabolic needs. However in recent years there were several reports that dogs may develop taurine deficiency-induced dilated cardiomyopathy (DCM). This has become more common in large dogs fed certain diets [2] and/or belonging to certain breeds [3, 4]. The main ingredients in the dog food fed to the taurine deficient dogs were lamb meal and rice, including rice bran [5]. Lamb meal has been reported to have a particularly low bioavailability of cysteine in dogs [6]. In a study where cats were fed a diet containing full fat stabilized rice bran, the cats had a lower blood taurine concentration compared to cats fed the same diet with cornstarch substituted for the rice bran [7]. Therefore, rice bran, in addition to lamb meal, may play a role in the development of taurine deficiency in dogs.

Various investigators have reported that the consumption of diets containing full fat rice bran results in reducing cholesterol concentrations in both liver and blood of several species; eg, in rats [8, 9], hamsters [10], and humans [11, 12]. One of the possible mechanisms for this effect is the high fermentability of rice bran, resulting in enhanced bile acid (BA) excretion and/or degradation (by increased microflora activity in the gut). According to the report of Gestel et al. [13], full fat rice bran fed to rats increased both BA excretion and bacterial activity, compared to controls fed a starch-based diet. Supporting evidence for the role of gut microflora in taurine loss has been reported for other species. Kim et al. [14, 15] reported a significant decrease in fecal cholyltaurine hydrolase activity (an enzyme produced by intestinal bacteria), and total fecal BA excretion with the addition of dietary antibiotics in cats. The administration of antibiotics resulted in the repletion of taurine in taurine-deficient cats within 3 weeks of treatment. It has been reported that dogs fed a lamb meal and rice diet showed higher urinary taurine excretion when antibiotics were added to their diet [16]. This suggests that highly populated microflora in the gut interferes with normal entero-hepatic re-utilization of taurine (taurocholic acid), which in turn prevents the maintenance of taurine homeostasis and decreases the quantity of taurine available for other metabolic functions and for renal excretion.

We postulate that dietary full-fat rice bran binds BA in the small intestine in dogs and thereby increases BA excretion, interfering with the entero-hepatic recycling of taurine-conjugated bile salts and lowering total body taurine status in dogs.

In the present study, the effects of dietary full fat rice bran on taurine status and BA excretion in dogs fed a diet near-limiting in sulfur amino acids were compared to those of dogs fed beet pulp, another common fiber source in dog food. Cellulose was used as the control fiber. Since an excess of dietary sulfur amino acids in dogs may mask the effects of marginally limiting sulfur amino acid metabolites such as taurine and glutathione, dogs were restricted in the amount of protein intake to 20–25 % above their minimum maintenance requirement described in National Research Council [17].

## Methods

### Animals and diets

The husbandry and treatments of the animals for the study were approved by the Animal Use and Care Administrative Advisory Committee at the University of California at Davis and the dogs in this study were taken care of in compliance with the National Research Council [18] guidance for laboratory animals. Eighteen intact male, mixed-breed dogs (Covance Stock and Broodstock Colony, Kalamazoo, MI, USA) were used for the study. Mean body weight (BW) of the 1–6 years old dogs at the initiation of the study was 29.1 ± 0.7 Kg (mean ± SEM). Six animals were assigned to each of the three experimental groups: cellulose (CL), beet pulp (BP) and rice bran (RB), based on similar BW of the dogs. The dogs were housed individually in indoor wire-mesh enclosures with coated rod-bottom floors at commercial facilities (Covance Research Products, Kalamazoo, MI, USA), providing a 12 h dark-light cycle and temperature control at 18–29 °C. Observations for general health and appearance were done three times a day at the discretion of the veterinarians and daily monitoring for food consumption was provided throughout the study. Weekly BW and body condition score (BCS, 9 point scale) were measured for each dog [19]. Food for all of the dogs for the study was provided once a day between 7 AM and 9 AM and water was given *ad-libitum* throughout the experiment.

Four complete, balanced diets were provided by a commercial laboratory animal food company (TestDiet®/LabDiet®, Purina, St. Louis, MI, USA). The ingredients and chemical compositions of the diets for the study are shown in Table 1. For the adaptation period, a pre-feeding (PF) diet, a complete and balanced dry expanded diet with 29.5 % protein containing 0.58 % methionine and 0.46 % cyst(e)ine (as-fed basis) was prepared to maintain an excess production of taurine for the maintenance of taurine homeostasis in these dogs. For the experimental period, three purified diets with the three different fiber sources, CL, BP and RB, were prepared, which, by design, included 12 % protein containing 0.23 % methionine and 0.12 % cyst(e)ine (as-fed basis). This prevented an excess of substrates for taurine synthesis that might overwhelm the effects of fibers on taurine metabolism studied. Twelve percent protein is higher than the minimum requirement of protein for maintenance of dogs described in National Research Council [17] and 0.35 % of sulfur amino acid concentration in the diets is within the range of total sulfur amino acid requirement (0.2–0.4 % of diets) for maintenance of adult dogs as determined by short-term nitrogen balance experiments [20–22]. Ten percent full-fat RB was used for the RB diet and the amounts of the fibers used in the other two diets were formulated, by calculation, to have the same amount of total dietary fiber (TDF) as that in the RB diet. Chromium oxide (0.02 %) was added to the experimental diets to use in determining apparent protein digestibility. The leftover and spilled food was collected daily and used to calculate food intake of each dog. The amounts of the food provided were adjusted weekly, based on changes of BW and BCS to aim toward an ideal BCS (5 out of 9 on the 9 point scale).

**Table 1:** Chemical composition and ingredients of the diets for the experiment^b,c^

	Units	PF Diet^a,d^	CL Diet^a^	BP Diet^a^	RB Diet^a^
Protein	%	29.5	11.8	11.7	11.7
Methionine + Cystine	%	1.04	0.35	0.35	0.34
Taurine	%	0.04	-	-	-
Fat	%	18.5	20.3	20.3	20.3
Fibers	%	2.0^e^	2.51^f^	1.98^f^	2.68^f^
Insoluble dietary fibers^f^	%	-	1.83	1.98	1.81
Soluble dietary fibers^f^	%	-	0.68	0.00	0.87
Metabolizable Energy^c^	kJ/g	15.0	19.2	18.7	17.5
Casein - vitamin free	%	-	5.00	6.71	3.00
Soy protein isolate	%	-	7.90	5.62	8.38
Corn starch	%	-	37.61	35.55	33.76
Sucrose	%	-	20.00	20.00	20.00
Lard	%	-	20.18	20.16	18.18
Cellulose – powdered^g^	%	-	2.15	-	-
Beet pulp– dried^g^	%	-	-	4.70	-
Rice bran – full fat^g^	%	-	-	-	10.00
Mineral/Vitamin^h^	%	-	6.84	6.94	6.36
Chromium oxide	%	-	0.02	0.02	0.02
Choline chloride	%	-	0.30	0.30	0.30
Total	%	-	100.00	100.00	100.00

Notes: ^a^ *PF* Pre-feeding, *CL* Cellulose, *BD* Beet pulp, *RB* Rice bran. ^b^The values were based on as-fed basis and provided from Purina Mills, LLC (St. Louis, MO) except where otherwise mentioned. All diets were formulated by the manufacturer to meet or exceed AAFCO (Association of American Feed Control Officials) requirements for macro and micronutrients for dogs. ^c^The values were calculated, based on the latest (as of March 2005) ingredient analysis information by Purina Mills, LLC (St. Louis, MO) except where otherwise mentioned. Since nutrient composition of natural ingredients varies, analysis will differ accordingly. ^d^PF diet; ingredients: ground corn, ground brown rice, poultry by-product meal, poultry meal, corn gluten meal, dehulled soybean meal, animal fat preserved with BHA (butylated hydroxyanisole), poultry fat preserved with ethoxyquin, wheat middlings, poultry digest, calcium carbonate, dried whole eggs, dried beet pulp, brewers dried yeast, soybean oil, dicalcium phosphate, salt, lecithin, pyridoxine hydrochloride, choline chloride, potassium chloride, menadione dimethylpyrimidinol bisulfate, biotin, cholecalciferol, vitamin A acetate, di-alpha tocopheryl acetate, inositol, DL-methionine, folic acid, calcium pantothenate, thiamin mononitrate, ethoxyquin, nicotinic acid, riboflavin, cyanocobalamin, manganous oxide, ferrous sulfate, cobalt carbonate, copper sulfate, zinc oxide, sodium selenite. ^e^Crude fiber. ^f^Total dietary fibers (TDF), dry-matter basis, analyzed by Dr. George C. Fahey Jr. in the Department of Animal Sciences, University of Illinois, Urbana, IL 61801. ^g^Amount of the ingredients was decided by calculation based on the amount of TDF equal to the amount of TDF in the rice bran diet with 10 % full fat rice bran. ^h^Provided the following amounts of minerals and vitamins/kg diet: calcium 10 g, phosphorous 6.6 g, potassium 7 g, magnesium 0.5 g, sodium 4.6 g, chloride 6.7 g, fluoride 48 mg, iron 365 mg, manganese 55 mg, copper 12 mg, cobalt 0.4 mg, iodine 1.5 mg, chromium 2.3 mg, molybdenum 1.23 mg, selenium 0.46 mg, vitamin A 10,900 IU, vitamin D-3 2,200 IU, vitamin E 44 IU, menadione 0.68 mg, thiamin hydrochloride 10.9 mg, riboflavin 4.9 mg, niacin 64 mg, pantothenic acid 20 mg, folic acid 4.0 mg, pyridoxine 12.4 mg, biotin 0.2 mg, vitamin B-12 28 μg, choline chloride 2.1 g

### Design and treatments

During the adaptation period the dogs were given the PF diet for 8 weeks to ensure that they were not taurine deficient. The last two weeks of the adaptation period were included for sample collections as week 0, which was the initiation of the measurements. Then, dogs were assigned to one of three experimental groups (CL, BP, or RB group, respectively) to establish similar mean BW among the experimental groups. During the experimental period (from week 2 to week 12), the three different experimental diets were given to the designated groups. Throughout the experiment, including the last two weeks of the adaptation period, blood was collected on the last day of each 2 week-period, urine was collected biweekly on a day before blood collection, and feces were collected during the last 5 days of each 2 week-period. Plasma (PL) taurine, whole blood (WB) taurine, urine taurine, BA excretion, apparent protein digestibility, blood thiols and PL complete amino acid profiles (CAAP) were measured. To assure the health conditions of the dogs, blood chemistries and complete blood cell counts were performed on the samples taken on the last day of the adaptation period (IDEXX Preclinical Research Services, Westbrook, ME, USA) and the concentrations of total protein and albumin of PL were done from the PL drawn on the last day of the experimental periods (Veterinary Medicine Teaching Hospital of the School of Veterinary Medicine, University of California, CA, USA).

### Sample collections and measurements

Blood (approximately 6 mL) was taken at the end of each 2 week-period to measure taurine, thiols, and amino acids. Every blood drawing was performed prior to feeding through cephalic vein by venipuncture using heparinized syringes (20 μL of sodium heparin solution, 1000 USP units/mL). Urine for assays of taurine and creatinine concentrations was collected, biweekly, for 14 h in an individual metabolic cage, one day before the blood was collected. The urine was collected in a container held in ice water.

A portion of WB (approximately 2 mL) was stored frozen (−20 °C) for WB taurine assay. Another portion of WB collected (approximately 4 mL) was immediately centrifuged (~1,200 × g for 10 min) to obtain PL. An aliquot of PL or urine (approximately 0.5 mL) was mixed with an equal volume of 0.24 mol/L of 5-sulfosalicylic acid, centrifuged at 15,800 × g for 15 min at 4 °C and the supernatant was collected. The resulting deproteinized PL or urine samples were assayed for taurine and for PL CAAP. The PL samples remaining were stored at −20 °C for determination of PL thiols. The frozen WB samples were thawed and frozen three times to lyse the blood cells, to release intracellular taurine, then diluted with an equal volume of double deionized water (DDIW) and deproteinized by the same method as described for PL.

Feces were stored at −20 °C. Each feces sample was mixed with DDIW to obtain a slurry homogenate. Approximately 100 g of the homogenate slurry of feces was frozen at −20 °C for BA and protein assay.

Taurine concentrations of deproteinized PL, WB and urine were measured using an amino acid analyzer (Beckman 7300 Analyzer C7 Model, Beckman Instruments, Fullerton, CA, USA). To normalize urinary taurine concentration, urinary creatinine concentrations were determined with a commercial kit (Cold Stable, Pointe Scientific Inc., Canton, MI, USA). Deproteinized PL CAAP was measured using an amino acid analyzer (Biochrom 30, Biochrom Ltd., Cambridge, UK). In order to quantify the concentrations of total cyst(e)ine (free plus that bound to protein), cysteinyl-glycine and homocysteine in PL and the concentrations of total glutathione (GSH + GSSG) in WB, the combined and modified HPLC method of Ubbink et al. [23] and Gilfix et al. [24] was used. All of the volumes of reagents and samples were scaled down to one-fourth to quantify thiol concentrations in PL and WB and, for WB total glutathione concentrations. WB blood was diluted with an equal volume of DDIW before assay. Bile acid concentrations in feces were measured using a commercial kit (Bile Acid Kit No. 450-A, Trinity Biotech USA, Jamestown, NY, USA) and BA in feces was extracted by the method of Porter et al. [25] Apparent digestibility of dietary protein was measured by calculation using dietary and fecal nitrogen concentrations. The concentrations of nitrogen, protein and chromium in the diets and feces were analyzed at Analytical Laboratory at the University of California Davis. Total nitrogen and total crude protein were measured by a nitrogen gas analyzer (LECO FP-528, LECO Corporation, St Joseph, MI, USA). Chromium concentration was determined by Inductively Coupled Plasma Atomic Emission Spectrometry (ICP-AES).

### Statistical analysis

Significance of the data among three experimental groups at each time point for all of the variables was analyzed by mixed regression. Comparison of the variables between two time points in a group was done by paired t-test. All data were analyzed using SAS program [26]. All data in the report were expressed as mean ± SEM unless otherwise mentioned. For all analyses, differences were considered significant at *P* < 0.05. Probability values in the range of 0.05≤ *P* < 0.1 were considered as an indicator of a noteworthy trend.

## Results

During the experimental period, one dog in the BP group ingested insufficient food to maintain BW and, therefore, was removed from the experiment at week 10 (BCS was 3 out of 9; BW was 82 % of week 0 at week 3). All of the data from the BP group were obtained from the remaining five dogs after week 10. Except for that dog, all of the dogs maintained BW and had normal blood chemistries and completed blood cell counts at the beginning of the experiment. However, at the end of the experiment, the mean PL albumin concentrations were 29 ± 2, 28 ± 1 and 29 ± 2 g/L for the CL, BP and RB group, respectively, which are below of the lower end of the reference range for PL albumin concentration of normal dogs (30–44 g/L). The mean PL total protein concentrations, at the end of the experiment, were 66 ± 4, 62 ± 5 and 66 ± 3 g/L for the CL, BP and RB group, respectively. These values are within the normal reference range for PL total protein concentration of normal dogs (54–76 g/L). There were no significant differences among the groups in PL albumin and total protein concentrations.

Mean food intakes (FI) of the dogs to maintain a BCS of 5/9 were 515 ± 35, 543 ± 43, and 566 ± 70 g/day for CL, BP and RB groups, respectively during the adaptation period and were 426 ± 11, 443 ± 14, and 425 ± 10 g/day for CL, BP and RB groups, respectively, during the experimental period. No significant differences among the groups were found throughout the study. Mean BW of the dogs at the end of the study were 24.8 ± 0.4, 27.1 ± 1.4 and 28.1 ± 1.3 kg for CL, BP and RB groups, respectively. No statistical differences in BW occurred among the groups during the experiment except that the CL group had lower BW than the RB group from week 9 to week 12 (*P* < 0.05). The BCS of all dogs were maintained between 4 and 6 throughout the study.

**Fig 1:**
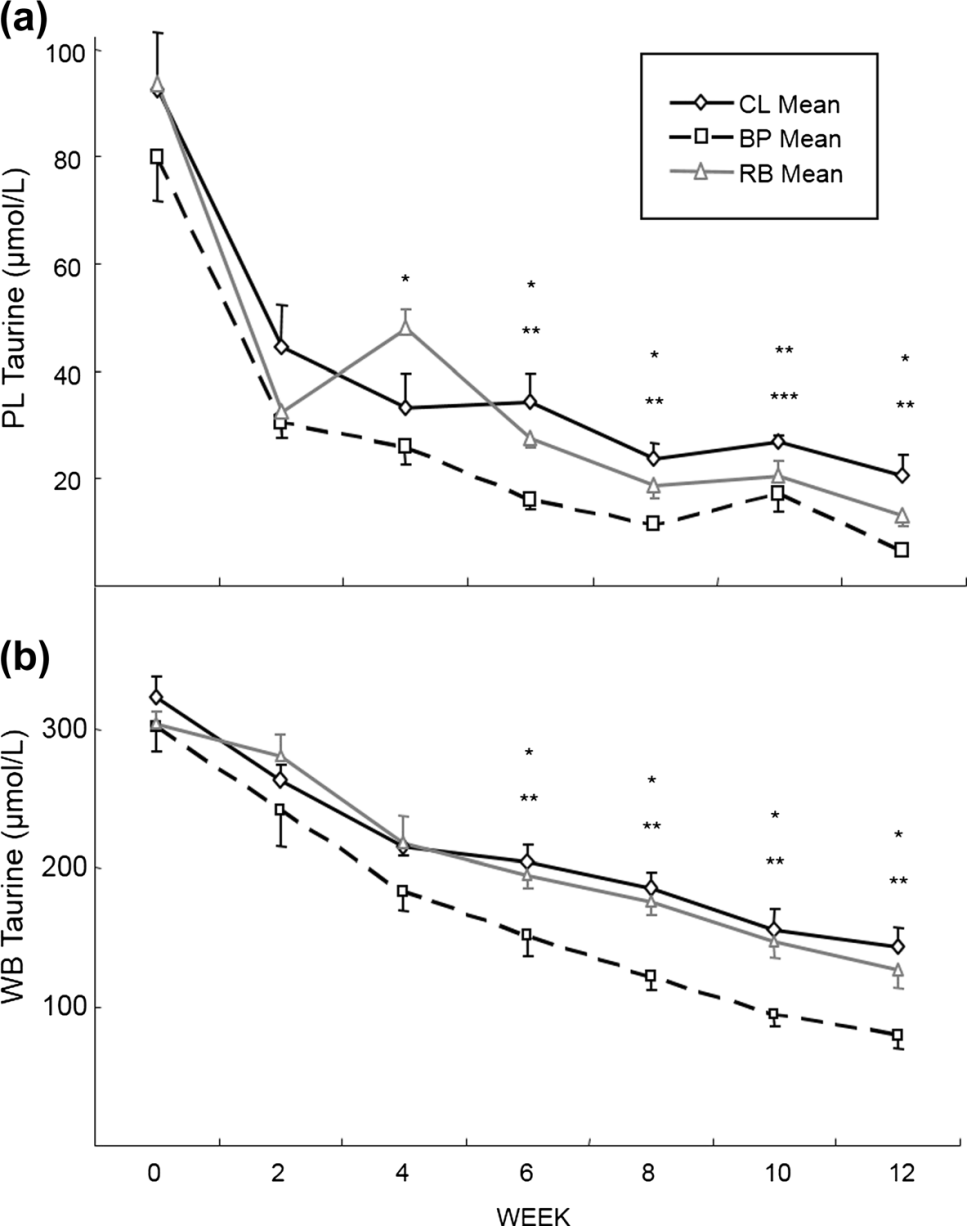
Concentrations of plasma (PL) and whole blood (WB) taurine among groups during 12 weeks of the experiment. **a** and **b** show taurine concentrations in PL and WB of dogs, respectively, fed the purified diets containing different fiber sources. The values are expressed as mean ± SEM. The symbols represent the groups that have significant differences (*P* < 0.05 unless otherwise mentioned in the text) at the time point (*significance between BP and RB, **significance between BP and CL, and ***significance between RB and CL). From week 10, *n* = 5 for the BP group due to omission of one dog for excessive weight loss

Plasma taurine concentrations decreased to under 40 μmol/L for all the groups (Fig. 1a) during the experimental period. The BP group decreased taurine concentrations lower than the other 2 groups from week 4 to the end of the experiment (*P* < 0.01) and the CL group maintained the highest mean PL taurine concentrations from week 6, but was significantly higher than the other two groups only at week 10 (*P* < 0.01). Whole blood taurine concentrations showed a similar pattern as those of PL taurine concentrations but the rates of decrease were slower (Fig. 1b). From week 6, the WB taurine concentration in BP group was lower than the other two groups (*P* < 0.01) with no statistical differences between the CL and RB groups. Urinary taurine excretions were markedly decreased from week 0; 3981 ± 790, 8880 ± 4496 and 5858 ± 910 nmol/ml/mg creatinine to week 12; 85 ± 7, 101 ± 23 and 120 ± 19 nmol/ml/mg creatinine for CL, BP and RB groups, respectively (ie, at week 12, only 2.1, 0.8 and 0.8 %, respectively of the week 0 values). However no statistical differences were found among the 3 groups at any time point.

Mean apparent protein digestibilities of the diets for the dogs were 88.8 ± 0.6, 81.9 ± 0.6 and 88.1 ± 1.2 % for CL, BP and RB groups, respectively. The BP group had the lowest protein digestibility from week 2 to week 12 (*P* < 0.001) and the RB group had a protein digestibility lower than the CL group but higher than the BP group at week 2, 4 and 8 (*P* < 0.05).

**Fig 2:**
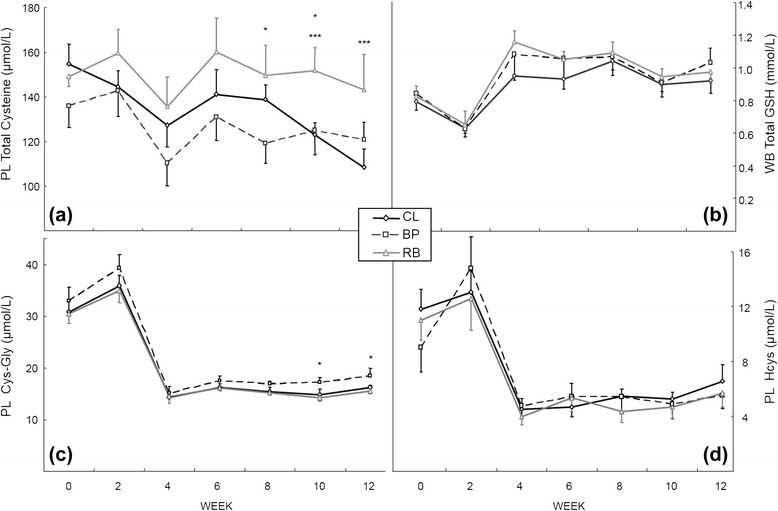
Various thiol concentrations in plasma (PL) and whole blood (WB) of the dogs fed the purified diets containing various fiber sources. **a** PL total cysteine (free + bound); **b** WB total glutathione (GSH + GSSG); **c** PL cysteinyl-glycine; **d** PL homocysteine. The values are expressed as mean ± SEM. The symbols represent the groups that have significant different (*P* < 0.05 unless otherwise mentioned in the text) at the time point (*significance between BP and RB and ***significance between RB and CL). From week 10, *n* = 5 for the BP group due to omission of one dog for excessive weight loss

The concentrations of thiols in PL or WB of the 3 groups are shown in Fig. 2. Plasma total cyst(e)ine concentrations (Fig. 2a) for RB group appeared to be maintained better than the other two groups which decreased during the experimental period. At week 8, the PL total cyst(e)ine concentration was significantly higher in the RB group than the BP group; at week 10, higher in the RB group than the BP and the CL group; and at week12, higher in the RB than the CL group (*P* < 0.05). Total glutathione (GSH + GSSG) in WB (Fig. 2b) did not show any significant differences among the groups. However, the mean concentrations of total glutathione in WB at week 12 of the CL, BP and RB groups increased by 46, 65, and 49 % from those at week 2 (*P* < 0.01). Plasma cysteinyl-glycine (Fig. 2c) and homocysteine (Fig. 2d) had similar patterns. The concentrations decreased between week 2 and week 4 and remained low until the end of the experiment. Only PL cysteinyl-glycine concentrations at weeks 10 and 12 between BP and RB, developed statistical differences (*P* < 0.05). The PL cysteinyl-glycine concentrations at week 12 were approximately 45, 47, and 45 % of those at week 2 (*P* < 0.01) and in PL homocysteine concentrations at week 12 were approximately 50, 37, and 45 % of those at week 2 (*P* < 0.01) for the CL, BP and RB group, respectively.

Plasma free cysteine (not including that bound to plasma proteins) and methionine concentrations were determined with the PL CAAP. At week 2, PL free cysteine in the BP group was significantly lower than the RB group (*P* < 0.05) with a noteworthy trend lower than the CL group (*p* = 0.08, 34 ± 3, 26 ± 4 and 36 ± 3 μmol/L for CL, BP and RB groups, respectively). However, at week 12, PL free cysteine concentrations among the two groups did not show any significant differences (23 ± 1, 25 ± 4 and 28 ± 5 μmol/L for CL, BP and RB groups, respectively). Plasma methionine concentrations did not show any significant differences among groups or with time. The mean PL methionine concentrations for CL, BP and RB group at week 0 were 53 ± 4, 56 ± 6 and 64 ± 7 μmol/L, respectively and at week 12 were 60 ± 2, 56 ± 2 and 51 ± 6 μmol/L, respectively.

Bile acid excretions of the dogs for the study are shown in Table 2. Throughout the study, the BP group had higher BA excretion than those of CL and RB regardless of the method of expression (*P* < 0.01).

**Table 2:** Bile acid excretion in dogs fed the purified diets containing various fiber sources^c^

	μmol/g feces (DM^e^-basis)			μmol/5 day		
Time	CL^e^	BP^e^	RB^e^	CL^e^	BP^e^	RB^e^
Week 0	5.17 ± 0.64^a^	7.03 ± 0.71^b^	5.76 ± 0.38^ab^	1123 ± 155	869 ± 120	1008 ± 67
Week 2	5.27 ± 0.62	6.56 ± 0.48	5.96 ± 0.21	944 ± 165	1145 ± 104	1047 ± 237
Week 4	4.53 ± 0.51^a^	7.40 ± 0.27^b^	5.17 ± 1.12^ab^	597 ± 119^a^	1053 ± 104^b^	721 ± 97^a^
Week 6	4.21 ± 0.63	4.18 ± 0.89	2.96 ± 0.39	684 ± 80	948 ± 241	514 ± 71
Week 8	2.69 ± 0.33^a^	5.34 ± 0.65^b^	4.93 ± 0.75^b^	487 ± 95^a^	868 ± 141^b^	693 ± 71^ab^
Week 10	3.32 ± 0.32^ab^	4.63 ± 0.84^a^	2.62 ± 0.34^b^	562 ± 92^ab^	^d^737 ± 146^a^	401 ± 52^b^
Week 12	3.43 ± 0.54^a^	5.60 ± 0.14^b^	3.42 ± 0.35^a^	513 ± 111^a^	^d^946 ± 177^b^	555 ± 64^a^

Notes: ^ab^The letters superscripted represent significant differences between groups (*P* < 0.05). ^c^The values are expressed as mean ± SEM, *n* = 6 for each group. ^d^ *n* = 5 due to omission of one dog for excessive weight loss. ^e^ *DM* Dry matter, *CL* Cellulose, *BP* Beet pulp, *RB* Rice bran

## Discussion

Food intake of the dogs decreased when the diets were changed from the PF diet to the experimental purified diets. Average FI of the dogs for the last two weeks of the adaptation period for the PF diet was 26 % higher than that for the experimental period for the purified diets. Decrease of FI after changing diets was mainly due to the characteristic differences of the diets. Purified diets have higher digestible energy as compared to the diets consisting of natural food ingredients. In this study, the energy (Table 1) of the PF diet (15.0 kJ/g diet) was about 20–25 % lower than those of the three purified diets (19.2 kJ/g diet, 18.7 kJ/g diet and 17.5 kJ/g diet for CL, BP and RB diet, respectively). However, the dogs throughout the study maintained BCS levels between 4 and 6. Food intake and protein intake throughout the study appeared to be adequate to maintain BW, even though the protein content of the purified diets was limited to about 12 % of their diets. However, the decrease in PL albumin below the normal reference range for all of three groups indicates that either total protein or an essential amino acid may have been slightly limiting for normal albumin homeostasis. Except for branched chain amino acids, none of the concentrations of essential amino acids in the PL during the experimental period were lower than those at week 0 (data not shown) and all were within the normal range for dogs [27]. The branched chain amino acids for weeks 8 and 12 were about 64–69 % of the concentrations found at week 0 (for PF diet) which were about at the first quartile of normal concentrations for dogs [27]. It would therefore appear that cyst(e)ine (60 % of the first quartile of the normal cyst(e)ine concentration) was the most limiting amino acid for protein synthesis as well as for taurine synthesis.

Mean taurine concentrations (Fig. 1) of the dogs at week 12 were 20.4 ± 3.9, 6.7 ± 0.5 and 13.1 ± 1.0 μmol/L for PL and 143 ± 14, 79 ± 10 and 127 ± 14 μmol/L for WB for the CL, BP and RB groups, respectively. Since the lower limits for PL and WB taurine concentration for preventing a risk for DCM in dogs are 40 μmol/L and 180 μmol/L, respectively [3], all of dogs that were participating in this study were taurine deficient by study’s end. There appears to be three reasons for the taurine deficiency in this study.

The first is protein digestibility (sulfur amino acid bioavailability). The BP group showed the lowest protein digestibility throughout the study among the three experimental groups and, therefore it would be predicted that less sulfur amino acids were available for taurine synthesis. The digestibility of protein in animals fed diets containing BP has been reported by various researchers. Several have reported no effect of BP on protein digestibility in horses [28], cats [29], and even in dogs [30]. In contrast, reports in pigs [31] and in chickens [32], indicate that protein digestibility was decreased when fed BP. It is known that taurine is a non-essential amino acid that is synthesized in most mammals from cyst(e)ine [33]. Our results indicate that when protein, and thus sulfur amino acids, are low yet sufficient for nitrogen balance and glutathione homeostasis, taurine synthesis is inadequate and that a decrease in protein digestibility may be a part of this process. Therefore, the key to a diet providing adequate taurine synthesis in dogs would be an adequate quantity of “bio-available” sulfur amino acids. That quantity appears to be more than we had in the diets of the present experiment.

The second possible reason is the effects of fibers that would interfere with the entero-hepatic recycling of BA, the recycling route for the major taurine metabolite to maintain taurine status. Fibers have various physiological effects on the metabolism of animals, including satiety, slowing gastric emptying, thus delaying or interfering with nutrient absorption that, in turn, results in improvement of glucose tolerance and lowering serum cholesterol [34]. There are several hypotheses regarding the cholesterol-lowering effect of fiber, which include increasing BA excretion through feces. Since most dogs diagnosed with DCM had been fed lamb and rice (including RB) diets, we postulated that RB may contribute to the low taurine status of these dogs [5]. In general, BAs are synthesized in liver from cholesterol and conjugated with glycine or taurine to make these strong detergents, glycocholic acid or taurocholic acid. These detergents play an important role in the small intestine to emulsify various kinds of lipids to enhance their absorption by forming water soluble micelles. After functioning, the bile salts are recycled via passive diffusion in the small intestine and via receptor-mediated transport in the lower ileum with approximately 99 % of recycling efficiency [33]. Dogs, like cats, obligatorily conjugate BA with taurine, ie, the liver enzyme responsible for conjugation, cholyl-CoA:*N-acyltransferase*, is specific for taurine in dogs [34]. An interference with entero-hepatic recycling of bile salts would result in the depletion of the taurine pool of dogs if a limited quantity of taurine or its precursors are available. Therefore, fecal BA excretion was determined as an indicator of the efficiency of entero-hepatic recycling of bile salts of the dogs fed the various fibers. Fecal BA excretions, on a dry matter basis, gradually decreased in all 3 groups (*P* < 0.01), apparently the result of switching from commercial diet to the purified diets. However, the excretion of BA/5 days by week 12 for the BP group was nearly twice that of the CL or RB group. Possible reason for decrease of fecal BA excretion with time may be the limited amount of protein in the diets. The synthesis and secretion of BA are reported to be enhanced by the hormonal stimulation of cholecystokinin whose release is evoked by fats and amino acids in the digestive tracks of the animals [35]. That is, the lower consumption of protein by the dogs may have led to less release of cholecystokinin and, in turn, less BA production and secretion. However, it is clear that the BP group had the highest BA excretion regardless of the method of expression. Even though the initial fecal BA excretion of the BP group on a dry matter basis was the highest, the BA excretions at week 12 were 64, 80, and 59 % of the BA excretions at week 0 for CL, BP and RB groups, respectively, showing that the BP group had the lowest percentage decrease. By analyses, the TDF of the CL, BP and RB diets (Table 1) were 2.51, 1.98, and 2.68 %, respectively, demonstrating that the BP fiber effect was not the result of more dietary fiber. Moreover, all had about the same percentage of insoluble dietary fiber, 1.83, 1.98 and 1.81 % for CL, BP and RB diets, respectively and all had about 1 % crude fiber. Thus, it does not appear that it is the quantity of the various fibers, but the nature of the fiber that is contributing to the different response of the BP on BA excretion and taurine depletion.

The third possible reason for the decrease in taurine status in the present study is the interaction of fiber with the small intestinal microbes. Thus, the difference between the overall effects of the three dietary treatments on taurine status may reside in the difference in fermentability of the fibers by the small intestinal microbes. If an increased microbial fermentation occurs as the result of an increased consumption of BP fiber as compared to the fiber in CL or RB, then it would be expected that more taurine would be destroyed, similar to the increase catabolism of taurine that occurs in cats that have more microbial fermentation [13–15, 36]. Sunvold et al. [37] have reported that BP supports a greater rate of fermentation than CL, and even if BP has no soluble fiber (ie, 100 % insoluble finer), it is still considerably more fermentable than CL. Thus, although we could not rule out some microbial fermentation by the CL or RB groups because we had no control diet without fiber, the results still support the idea that BP, not RB, fiber may contribute to a significant loss of endogenous taurine in dogs.

With the possible exception of free and total PL cyst(e)ine, there is no indication that there was an effect of fiber on body thiol status (Fig. 2). Although total PL cyst(e)ine (free + bound) was somewhat lower in the BP group at 4 of the time points, it was never significantly lower than the CL group, suggesting that it was not a decrease in PL cyst(e)ine alone that was the cause of the lower PL taurine in the BP group, even though it is apparent that there was not enough dietary sulfur amino acids (more specifically, hepatic cysteine) for any group to synthesize adequate taurine. Plasma free cysteine (cysteine not bound to protein) concentrations in the current experiment were already depleted in the BP group (26 ± 4 μmol/L) at week 2 and were maintained until the end of the study (25 ± 4 μmol/L), whereas the other 2 groups, although decreasing at week 2 (34 ± 3 μmol/L and 36 ± 3 μmol/L for CL and RB group, respectively), were not as depleted as the BP group until week 12 (23 ± 1 μmol/L and 28 ± 5 μmol/L for CL and RB group, respectively) supporting the idea that dietary BP decreases the bioavailability of cysteine in the diet, thus contributing to the depletion of taurine in dogs.

Whole blood was chosen for total glutathione assay since red blood cells contain the higher concentration of glutathione. Although glutathione is known as a reservoir for cysteine [38], it is interesting that in the present experiment glutathione did not decrease after feeding the low protein diets but actually increased about 20 % in all groups. According to Stipanuk et al. [38] glutathione concentration is regulated by the activity of glutamate-cysteine ligase (known as γ-glutamyl-cysteine synthetase) whose activity is regulated by the cellular concentration of cysteine. When cellular cysteine is decreased, glutamate-cysteine ligase is up-regulated to increase synthesis of glutathione and when cellular cysteine is in excess, cysteine dioxygenase is up-regulated to catabolize excess cysteine to maintain a narrow range of tissue cysteine concentrations. In this study, the concentrations of total cysteine in PL at week 12 were approximately 75, 85 and 90 % of those of week 2 for CL, BP and RB group, respectively. Free cysteine concentrations in PL at the end of the study were 62, 49, and 46 % of those of week 0 for CL, BP and RB group, respectively, perhaps indicating that free cyst(e)ine is a better indicator of cysteine availability for metabolic needs (including taurine synthesis) than total cyst(e)ine (free plus that bound to protein via sulfhydryl bonding).

It is interesting that metabolic regulation conserves glutathione rather than taurine, perhaps simply because the dietary excess of sulfur amino acids goes through the liver first where the majority of the enzymes involved are located and because of the *Km*s of the enzymes involved. That is, the priority for the use of cysteine in dogs in our experiment appears to be first for glutathione, second for general protein synthesis and finally for taurine synthesis. The apparent anomaly (ie, of glutathione being a reservoir for cyst(e)ine) here is that there appeared to be insufficient albumin synthesis or increased albumin breakdown under the conditions of our experiment, even though the dogs appeared to be in nitrogen balance (ie, maintaining BW) and WB total glutathione actually increased.

## Conclusion

In summary, rather than RB, dietary BP showed the most significant effect in lowering PL and WB taurine concentrations, in part, by decreasing the protein digestibility (sulfur amino acid bioavailability), by enhancing fecal excretion of BA and possibly, by enhancing degradation of taurine by gut microflora in dogs. These effects may result from the greater effect of BP fiber than RB or CL on intestinal bacterial fermentation that cleaves taurocholic acid and destroys the taurine released. In conclusion, since CL was the control fiber, and RB caused similar responses as CL, we conclude that RB is unlikely the cause of the increased risk of taurine deficiency in dogs fed lamb and rice diets.
